# Subjective Cognitive Decline and Genetic Propensity for Dementia beyond Apolipoprotein ε_4_: A Systematic Review

**DOI:** 10.3390/cimb46030129

**Published:** 2024-03-02

**Authors:** Stefanos N. Sampatakakis, Maria Roma, Nikolaos Scarmeas

**Affiliations:** 1 1st Department of Neurology, Aiginition Hospital, Athens Medical School, National and Kapodistrian University, 11528 Athens, Greece; stefanos.sab@gmail.com (S.N.S.); rwma.maria@gmail.com (M.R.); 2 Department of Neurology, The Gertrude H. Sergievsky Center, Taub Institute for Research in Alzheimer’s Disease and the Aging Brain, Columbia University, New York, NY 10027, USA

**Keywords:** subjective cognitive decline, cognitive complaints, early dementia stages, genetic propensity, polygenic risk

## Abstract

Subjective cognitive decline (SCD) has been described as a probable early stage of dementia, as it has consistently appeared to precede the onset of objective cognitive impairment. SCD is related to many risk factors, including genetic predisposition for dementia. The Apolipoprotein (APOE) ε4 allele, which has been thoroughly studied, seems to explain genetic risk for SCD only partially. Therefore, we aimed to summarize existing data regarding genetic factors related to SCD, beyond APOE ε4, in order to improve our current understanding of SCD. We conducted a PRISMA systematic search in PubMed/MEDLINE and Embase databases using the keywords “subjective cognitive decline” and “genetic predisposition” with specific inclusion and exclusion criteria. From the 270 articles identified, 16 were finally included for the qualitative analysis. Family history of Alzheimer’s disease (AD) in regard to SCD was explored in eight studies, with conflicting results. Other genes implicated in SCD, beyond APOE ε4, were investigated in six studies, which were not strong enough to provide clear conclusions. Very few data have been published regarding the association of polygenic risk for AD and SCD. Thus, many more genes related to AD must be studied, with polygenic risk scores appearing to be really promising for future investigation.

## 1. Introduction

Nowadays, awareness of brain health and symptoms of dementia in the general population is growing. Therefore, an increasing number of cognitively unimpaired individuals seek medical help for their cognitive function [1]. A new medical term, subjective cognitive decline (SCD), was conceived in 2014 in order to describe an individual’s perception of their own cognitive impairment [2]. At the same time, the SCD initiative (SCD-I), an international working group, established standardized criteria for SCD [2], consisting of a self-experienced persistent decline in cognition, unrelated to an acute event, in comparison to a previously normal cognitive status, along with normal performance on standardized cognitive tests.

Existing data suggest that SCD may precede the onset of objective memory impairment [3], acting as an early dementia stage [4]. Specifically, longitudinal studies have shown that individuals reporting SCD had an increased incidence of dementia [5,6]. Moreover, cognitively normal (CN) individuals with SCD have been related to abnormal Alzheimer’s disease (AD) biomarkers, such as low cerebrospinal fluid amyloid-beta [7,8], as well as low hippocampal and medial temporal cortex volume [9,10]. Individuals with SCD have also been associated with peripheral blood biomarkers [11] of AD, such as the peripheral blood transcriptome, which might be considered a pre-disease biomarker.

However, it remains uncertain which factors affect the progression from SCD to dementia. In fact, SCD has been related to many different underlying causes, apart from preclinical dementia, including psychiatric symptoms or disorders [12], medication effects [13], different personality traits [14], poor physical functioning and alcohol abuse [15]. Additionally, the context (community-based or clinical setting) [16] as well as the way SCD is assessed (via a single question or structured questionnaire) [17] have been shown to affect its prevalence. Thus, individuals with SCD appear to be a highly heterogeneous population. Given that, to date, there is no single gold standard instrument which can differentiate individuals with SCD from those without SCD in the clinical setting; SCD is not considered a diagnostic entity in the Diagnostic and Statistical Manual of Mental Disorders [18]. What is of utmost importance, though, is to identify those individuals belonging in the predementia spectrum in whom SCD may yield additional information for future objective cognitive decline [19].

In an effort to determine the factors increasing the risk of an individual with SCD to develop objective cognitive impairment, the SCD-I working group has proposed the SCD-plus criteria [2]. The specific criteria focused on memory rather than other cognitive domains. Moreover, confirmation of perceived cognitive decline by an informant was included, in accordance with previous studies showing that partner reports of memory impairment were more consistent in estimating objective impairment and predicting longitudinal decline [20,21]. Interestingly, Apolipoprotein (APOE) ε4 carriership, which is most commonly associated with late onset AD [22], was also included in the SCD-plus criteria, enhancing the hypothesis of an association between SCD and genetic predisposition for dementia. Thus, genetic risk factors for AD might be helpful in distinguishing individuals with SCD with a higher risk for dementia.

To date, the vast majority of studies regarding genetic predisposition for dementia in individuals with SCD have compared APOE ε4 carriers with non-carriers. The APOE ε4 allele has been associated with cognitive decline [23], abnormal levels of amyloid and tau proteins [24], cortical atrophy [25], disruption of the cerebral white matter in neuroimaging [26,27] and functional brain network changes [28] in individuals with SCD. A previously published systematic review summarized existing data regarding the contribution of APOE ε4 in SCD [29], including 36 articles published between 2001 and 2018, and concluded that there is not enough evidence to suggest that the specific allele predisposes individuals to developing SCD. Recent research work has indicated that APOE ε4 allele is not the only genetic factor implicated in objective cognitive decline among individuals with SCD [30]. Moreover, in the FACEHBI study [31], the APOE genotype has been shown to explain the variance of cerebral amyloid levels only partially in individuals with SCD. Those findings have aroused interest in the possible involvement of other genetic factors for dementia, beyond APOE ε4, in SCD.

The genetic profile of individuals with SCD, beyond APOE ε4, is relatively unexplored. Family history (FH) of AD has been related to increased worrying about dementia symptoms [32], as well as increased risk of developing dementia [33]. Thus, family studies investigating siblings or individuals with positive family history (FH) of AD in relation to SCD have been conducted. Furthermore, other genes beyond the APOE ε4 allele have appeared to contribute to SCD, including presenilin-1 (PSEN1) gene mutations [34,35] as well as period circadian clock 2 (PER_2_) gene polymorphisms [36]. Nevertheless, only a small proportion of relevant genes has been studied. Additionally, the polygenic risk for AD has recently emerged, with research concerning the polygenic risk for SCD being relatively limited.

Therefore, our aim in undertaking the current systematic review was to summarize existing knowledge concerning the association of genetic propensity for dementia, beyond APOE ε4, and SCD, as well as to detect whether such an association might be used in order to ameliorate screening for future decline and identify effective interventions for dementia risk reduction.

## 2. Methods

### 2.1. Systematic Review Search Strategy

The systematic review was conducted in accordance with the Preferred Reporting Items for Systematic Review and Meta-analysis Protocols (PRISMA) statement [37]. The PRISMA 2020 checklist can be found in Table S2 of the Supplementary Materials, along with the PRISMA 2020 checklist for abstracts (Table S3). The protocol of the systematic review was registered in PROSPERO (ID: CRD42024507607).

PubMed/MEDLINE and EMBASE, two electronic bibliographic databases, were searched from inception until 15 December 2023. The search term “subjective cognitive decline” or “subjective cognitive complaints” or “subjective memory complaints” was used in combination with the terms “genetic predisposition” or “genetic risk” or “genetic factors” or “polygenic risk”. Overall, 366 records were identified. The exact search strings of the systematic review search strategy can be found in the Supplementary Materials.

**Figure 1:**
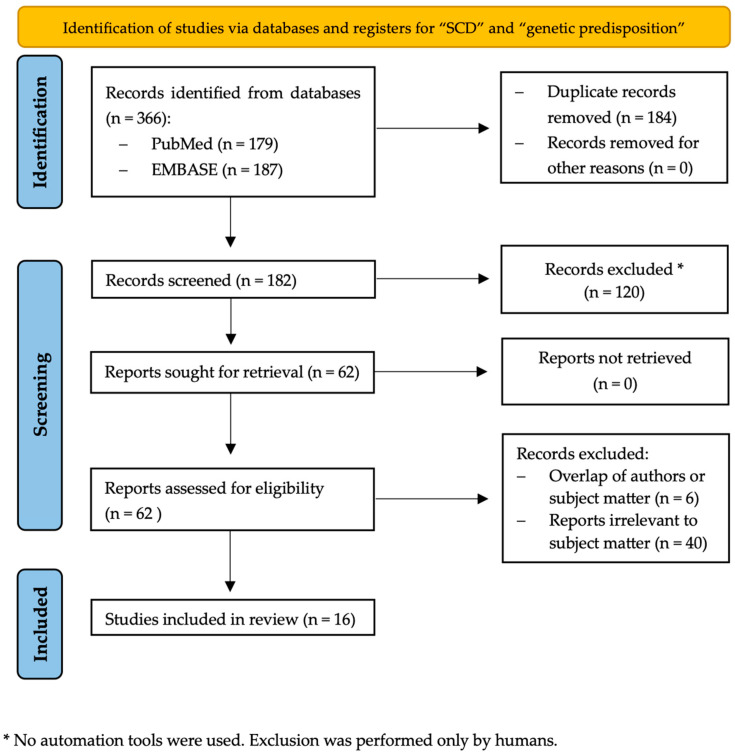
PRISMA flow diagram of systematic review.

The different phases of the systematic review performed are described in Figure 1.

### 2.2. Screening and Eligibility Strategy

The screening and eligibility phase was blindly conducted by two individual researchers (S.N.S. and M.R.). All titles and abstracts retrieved from the search were independently examined by the researchers on the same day to promote reliability. After removing 184 duplicate records, the first screening of the remaining 182 reports resulted in 62 full articles. Inclusion criteria were the following: full-text original peer-reviewed research articles (abstracts of poster presentations available in the bibliographic databases were not included), study population including a proportion of individuals with subjective cognitive complaints (SCC) or subjective memory complaints (SMC) and English language. We mainly selected publications from the past ten years but did not exclude commonly referenced and highly regarded older publications. In total, 120 records were excluded based on the exclusion criteria presented in detail in Table S1 of the Supplementary Materials.

The 62 full-text articles identified as relevant during the first screening stage were assessed for eligibility. In particular, studies were evaluated for potential overlap of authors and subject matter, leading to exclusion of 6 more reports. As this specific review concerns the relationship between SCD and genetic predisposition for dementia beyond APOE ε4, articles examining only the APOE ε4 allele were excluded as irrelevant to subject matter (40 reports), leading to 16 studies which were finally included. In case of disagreement during the eligibility assessment, another investigator (NS) reviewed the full text in question and gave a final approval.

## 3. Results

In total, 16 studies were selected for the final stage and qualitative synthesis to achieve the objectives of this systematical review. The results were stratified into “family studies” including studies concerning family history (FH) of AD as well as studies in twin individuals, “other genes beyond APOE ε4”, and “polygenic risk”. In all studies, SCD was assessed in the context of reported SMC via single questions or structured questionnaires.

### 3.1. Family Studies

In total, eight family studies were included [38,39,40,41,42,43,44,45], either relating FH of AD to SCD (five studies) or examining SCD heritability in twin individuals (three studies). Family studies are shown in Table 1.

FH studies have produced conflicting results regarding SMC. On the one hand, Nicholas et al. [38] did not find an association between FH and baseline or longitudinal SMC in 1545 CN subjects, among whom 72.4% reported a parental FH of AD. Similarly, in the case–control study of Heun et al. [39] including 550 individuals (238 first-degree relatives, 59 spouses of AD patients and 253 controls), SMC were not associated with FH of AD, as no difference was found between relatives, spouses and controls. In contrast, Heser et al. [40] found that SMC were associated with FH of AD (*p* = 0.008) in a prospective multicenter study including 1240 individuals without dementia, among whom 422 had a parental FH of AD. Additionally, FH of AD was associated with subsequent risk of all-type dementia in participants with SMC [hazard ratio (HR): 1.56, 95% confidence intervals (CI): 1.18–2.07, *p* = 0.002)]. These findings are in accordance with the case–control study of Tsai et al. [41], which showed that first-degree relatives were more likely to report SMC than spouses of AD patients [odds ratio (OR): 1.9, 95% CI: 1.3–3.0]. Moreover, another case–control study conducted by Haussmann et al. [42] investigating 40 CN and 35 mild cognitive impairment (MCI) individuals, showed that SMC were greater in participants with FH of AD only in the CN group (*p* = 0.019).

In all twin studies included in this systematic review, SMC were not related to genetic background. In particular, a longitudinal study conducted by Selwood et al. [43] included 338 monozygotic and 274 dizygotic twin pairs without dementia, revealing that SMC were low to moderately heritable, as just one third (33%) of the SMC variance was explained by genetic factors, while environmental factors were responsible for 67% of the SMC variance. Similar results were obtained by Bell et al. [44] who did not find an association between SMC and parental FH of AD in 1.555 twin male individuals studied prospectively, showing that the heritability for SMC ranged from 26% to 34% in males aged 38–67. Last but not least, Caracciolo et al. [45] assessed 11.926 twin individuals from the large Swedish cohort study for SMC via a telephone interview, concluding that SMC occurrence was not related to genetic background.

### 3.2. Other Genes beyond APOE ε4

Overall, six studies [34,35,36,46,47,48] regarding other genes, beyond APOE ε4, were included in this specific review, as presented in Table 2.

Τwo studies investigated different PSEN-1 mutations. The E280A mutation was studied in 52 individuals who all had a parent who was carrier of the specific mutation. Among participants, 26 were carriers themselves, while 26 were non carriers [34]. In that study, self-reported SMC were higher in carriers of the mutation (*p* = 0.019), while partner-based SMC did not differ between carriers and non-carriers. Furthermore, the PSEN-1 Glu318Gly mutation was investigated in a case–control study of Australian individuals, including participants with SMC [35]. The specific mutation was found only in four individuals in the SMC group, while no-one among controls had the mutation. Although Glu318Gly carriers had lower objective cognitive scores, SMC did not differ between carriers and non-carriers.

Circadian CLOCK and PER genes polymorphisms, implicated in a sleep–wake cycle, were investigated by Bessi et al. [36] in a longitudinal study. CLOCK T3111C polymorphism was detected in 51.2% of cases with SMC, while PER2 C111G polymorphism was found in 19.5% of cases with SMC. During follow-up, only two subjects with SMC progressed to AD (all were PER2 G carriers), while none of the SMC non-carriers converted to AD (*p* = 0.003).

The KIBRA (kidney and brain expressed protein) T allele, mainly expressed in memory-related brain regions, was investigated in a prospective study conducted by Mazzeo et al. [46] with a long follow-up period. Among 70 individuals with SCD, 16 developed MCI at follow-up [11 were carriers of one or two T alleles (CT/TT) and 5 were not (CC)]. Carriers of the T allele (CT or TT) had 2.273 times higher odds of MCI development than CC carriers (*p* = 0.049) at follow-up.

The APOE ε7 allele, a mutant form of APOE ε3, was investigated in a case–control study by Youn et al. [47], in which ε3/ε7 heterozygotes were found only in the SMC group of individuals. Lastly, Watfa et al. [48] examined the contribution of 50 different gene polymorphisms associated with vascular alterations in dementia-free participants with hypertension and SMC at the time of investigation. No significant associations were found for the genes investigated.

### 3.3. Polygenic Risk and SCD

Data regarding the association of polygenic risk for AD and SCD are very limited as only three relevant studies have been published [44,49,50], shown in Table 3.

A study conducted by Ebenau et al. [49] examined the effect of a polygenic risk score (PRS) predicting AD in a population of cognitively normal subjects with SCD from the Amsterdam Dementia Cohort. The specific PRS was calculated based on 39 genetic variants previously shown to be related to AD [51,52,53], excluding APOE, and was associated with amyloid positivity, according to the ATN classification (OR: 1.5, 95% CI 1.2–2.0), in subjects with SCD. In addition, a higher PRS increased AD risk (HR: 1.7, 95% CI 1.1–2.8), while individuals with a higher PRS had a lower risk of non-AD dementia (HR: 0.5, 95% CI 0.3–0.9). Nevertheless, after adjustment for APOE, the relationship between the PRS and progression to AD was not significant anymore.

Another study conducted by Sathyan et al. [50] examined the polygenic inheritance of SCD in the context of motoric cognitive risk syndrome, a predementia syndrome characterized by the presence of SMC and slow gait. In that case, the existence of SMC in older individuals was not associated with the PRS related to AD, including 19 genetic loci from previous GWAS studies [54]. However, increased neuroticism PRS was associated with SMC (OR: 1.10, *p* = 0.007). The same PRS for AD was also used in the aforementioned twin study conducted by Bell et al. [44], in which it was not associated with SMC in a statistically significant manner.

## 4. Discussion

In our systematic review, we sought to summarize existing evidence on AD genetic factors beyond APOE ε4 in regard to SCD. We found that family studies, both FH and twin studies, have produced contradictory results regarding the association of interest. Moreover, studies of specific gene alleles, apart from PSEN1, including KIBRA T, PER2 and APOE ε7 cannot establish a direct association with SCD. Last but not least, the polygenic risk for AD, in the context of PRSs, has not yet been thoroughly studied to provide clear conclusions.

### 4.1. Family Studies

To begin with, FH of AD was studied using first-degree relatives of patients with AD as a composite risk factor for dementia, as shown previously [55]. FH studies have yielded conflicting results regarding the association of FH of AD and SMC. On the one hand, the longitudinal study of Nicholas et al. [38] as well as the case–control study of Heun et al. [39] showed that SMC were not associated with FH of AD. On the other hand, Tsai et al. [41] as well as Haussman et al. [42] found that SMC were related to FH of AD in all participants and in the CN group, respectively. The most recent multicenter study of Heser et al. [40] showed a strong association between SMC and FH of AD (*p* = 0.008), as well as increased all-type dementia risk (*p* = 0.002). In that study, the authors considered a negative participant’s answer valid regarding FH only if the respective maternal age or age of death was at least 75 years (or paternal age at least 70 years), resulting in a very high missing data rating for FH (approximately 60%). Additionally, the mean age of participants was approximately 80 years old; hence, the results may not apply to younger cohorts.

Selection bias is the most important limitation of FH studies, as some participants were excluded due to absence of living relatives [40], while self-selection may have resulted in a healthier sample with fewer memory complaints than the general population [41]. In any case, the generalizability of results is limited. Moreover, SCD estimation in most family studies was made using simple questions, either via in-person or telephone interviews, except for one study [38], in which a structured SMC questionnaire was used. Thus, the SMC assessment raises questions concerning the reliability of the results, as simple questions are likely to be vague and, therefore, hide useful information, leading to an increased risk of misclassification. Last but not least, an important concern regarding FH studies, compared to single genetic variants such as APOE ε4, is the possible contribution of the psychological distress caused by the knowledge of being at increased risk of SCD. There is an ongoing debate about whether disclosing genetic dementia risk information has negative psychological consequences on relatives of patients with AD [56].

All twin studies [43,44,45] concluded that there was no association between SCD and genetic background. However, questions arise regarding the generalizability of these results in different populations, as the ethnic background of participants was limited, and all studies included community-dwelling populations, while one study included only male individuals [44]. The twin study design itself offers an advantage, allowing researchers to differentiate between the contributions of genetic and environmental factors [43]; thus, the conduction of multi-ethnic, larger twin studies in the future is essential.

### 4.2. Other Genes beyond APOE ε4

As far as other alleles beyond APOE ε4 are concerned, only PSEN1 280A mutation carriers were cross-sectionally related to SCD [34]. PSEN1 280A mutation, which is recognized as the most common cause of familial early-onset AD with complete penetrance, meaning that eventually all carriers will develop AD until their fifth decade of age, appears to be associated with SCD as well. Prospectively, KIBRA T allele [46], which is involved in cell migration and synaptogenesis, as well as PER2 G carriership [36], which is involved in sleep–wake cycle alterations and neurodegeneration, were related to increased longitudinal cognitive decline in subjects with SCD; however, the number of events was relatively small. Among the rest of the gene variants studied [35,47], neither the APOE ε7 allele nor the PSEN-1 Glu318Gly mutation were directly associated with SCD and longitudinal cognitive decline, even though these were found only in the SMC groups. The 50 genes related to vascular damage [48] were not associated with cognitive decline in subjects with SMC receiving anti-hypertensive treatment, similarly to inflammatory genes studied in the past [57].

The aforementioned studies offered indications rather than conclusions concerning the genetic profile of individuals with SMC, presenting remarkable limitations, such as the generalizability of the findings due to the specific features and characteristics of the population samples and the statistical power being limited by the relatively short sample sizes. Additionally, each study used a different questionnaire to evaluate SMC, enhancing the heterogeneity of participants’ answers. A recent study aimed to link questionnaire data from international aging studies and was able to differentiate items that made the greatest contribution to measurement precision, opening new perspectives of developing new self-perceived cognitive functioning questionnaires [58]. In any case, many more genes need to be investigated, such as the bridging integrator 1 (BIN_1_), clusterin (CLU), and phosphatidylinositol-binding clathrin assembly protein (PICALM), which have been previously shown to be associated with increased risk for late onset AD [54] and also predict objective verbal memory (*p* < 0.05) [59].

### 4.3. Polygenic Risk

To date, polygenic risk in subjects with SCD has not been studied in depth. Only in one study [49] higher PRS for AD was associated with increased amyloid positivity as well as progression to AD in a population with SCD (an association which was no longer significant after adjusting for APOE), while the other two studies [44,50] which used a different PRS for AD did not show an association. Another study, not included in our results due to the unavailability of the full text, examined the application of a PRS algorithm (genoSCORE), including over 100.000 genetic variations related to AD, in subjects with SCD and MCI in a London Memory Clinic [60], with promising results, as individuals with SCD who were most likely to decline cognitively towards AD had higher PRS values. Taking the above-mentioned results [44,49,50] into consideration, it appears that a variety of different genes is implicated in SCD, just like AD [61]; thus, the PRS method can be helpful in future studies in revealing the genetic underpinnings of SCD.

### 4.4. Limitations of Our Study

This systematic review is not without limitations. First of all, data regarding SCD and genetic predisposition for dementia, beyond APOE ε4, are heterogeneous. Study populations were heterogeneous in terms of age, cognitive status (including CN, MCI or dementia-free participants) and ethnicity. Additionally, SCD was not uniformly defined in included studies. SCD assessments were performed based on different questionnaires, which may in turn lead to differences in outcomes. As we mentioned before, efforts have already been made in order to combine existing questionnaires into one reliable and standardized questionnaire concerning self-perceived cognitive functioning [58]. The systematic review strategy as well as adequate blinding of researchers have mitigated selection bias. However, selection bias might anyway exist (might be attributed to several exclusion criteria such as year of publication or language of the text). Nevertheless, the overall outcomes of this review provide a comprehensive overview of existing knowledge.

## 5. Conclusions

Individuals with SCD appear to be a genetically heterogeneous population. The APOE genotype represents only a small proportion of the genetic propensity for SCD. The contribution of FH of AD to SCD remains controversial, as conducted studies present remarkable limitations in terms of selection bias, SMC assessment and probable psychological effects of FH; thus, many more twin studies should be performed. Apart from the PSEN1 280A mutation, studies concerning other candidate genetic variants such as KIBRA T, PER2 and APOE ε7 offer only indications of a possible association with SCD; therefore, many more genes associated with AD must be investigated in relation to SMC. In the near future, the application of the PRS method, which has already shown promising results, has the potential to contribute towards management planning and might be used in individualized risk profiling for longitudinal cognitive decline in subjects with SCD.

## Figures and Tables

**Table 1:** Family Studies in subjects with SMC.

Study	Population	Family History	SMC ^1^ Estimation	Main Results
Nicholas et al., 2017 [38] Longitudinal	Ν = 1545, United States CN ^2^ Mean age: 53.6	1118 individuals with parental FH ^3^ of AD ^4^	MFQ: Memory Functioning Questionnaire	SMC (baseline or longitudinal) were not associated with FH
Heun et al., 2003 [39] Case-Control	N = 550, Germany CN, MCI ^5^ or dementia Mean age: 67.9 ± 10.2	238 individuals with parental FH of AD, 59 spouses	In-person interview with two questions	SMC were not associated with FH of AD
Heser et al., 2023 [40] Longitudinal	N = 1240, Germany Dementia-free Mean age: 79.6	422 individuals with parental FH of AD	In-person interview with two questions	SMC were associated with FH of AD (*p* = 0.008). FH was related to increased dementia risk (HR ^6^: 1.56, *p* = 0.002) in SMC participants
Tsai et al., 2006 [41] Case-Control	Ν = 1499, United States and Germany, CN Mean age: 69.6 ± 10.5	1203 individuals with parental FH of AD, 296 spouses	In-person interview with a single question	SMC were greater in participants with FH of AD (OR ^7^: 1.9, 95% CI ^8^: 1.3, 3.0)
Haussmann et al., 2018 [42] Case-control	N = 75, Germany CN (40) or MCI (35) Mean age: 68.1 ± 7.1	21 individuals with parental FH of AD	7-item Likert scale (1 = severe to 7 = none)	SMC were greater in participants with FH of AD in the CN group (*p* = 0.019)
Selwood et al., 2017 [43] Longitudinal	N = 612, Australia Dementia-free Mean age: 71.3 ± 5.7	Twin individuals 338 monozygotic 274 dizygotic twins	Telephone interview with two questions	SMC were low to moderately heritable (h^2 9^ = 0.33, CI: 0.15, 0.49)
Caracciolo et al., 2012 [45] Longitudinal	N = 11,926, Sweden Dementia-free Aged ≥ 65	Twin individuals	Telephone interview with specific questions	SMC were not related to genetic background
Bell et al., 2023 [44] Longitudinal	N = 1555, United States Male adults Mean age: 37.8 ± 2.5	Twin individuals 872 monozygotic 570 dizygotic twins	In-person interview with a single question	SMC were not related to FH of AD

^1^ Subjective memory complaints, ^2^ cognitively normal, ^3^ family history, ^4^ Alzheimer’s disease, ^5^ mild cognitive impairment, ^6^ hazard ratio, ^7^ odds ratio, ^8^ confidence interval, ^9^ heritability.

**Table 2:** Studies concerning other genes beyond APOE ε4.

Study	Population	Genes	SMC ^1^ Estimation	Main Results
Norton et al. [34], 2017 Cross-Sectional	N = 52, Colombia CN ^2^, 26 carriers Mean age: 36.4 ± 7.1	PSEN-1 280A mutation	Self-reported and partner-based with Memory Complaint Scale	Only self-reported SMC were higher in carriers (*p* = 0.019).
Laws et al. [35], 2002 Case-Control	Ν = 124, Australia 58 SMC individuals Mean age: 62.0 ± 1.8	PSEN-1 Glu318Gly mutation	Cambridge Cognition Examination (CAMCOG)	Only 4 SMC subjects (6.8%) had the mutation. No difference in SMC.
Bessi et al. [36], 2021 Longitudinal	N = 68, Italy 41 SCD ^3^, 27 MCI ^4^ Mean age: 65.2 ± 7.2	CLOCK _T3111C_ PER2 _C111G_	Self-reported with a single question	Only 2 SCD subjects prοgressed to AD (all were PER2 G carriers, *p* = 0.003).
Mazzeo et al. [46], 2017 Longitudinal	N = 101, Italy 70 SCD, 31 MCI Mean age: 61.3 ± 7.9	KIBRA T	Memory Assessment Clinics-Questionnaire (MAC-Q)	CT or TT carriers had 2.273 higher odds of MCI than CC carriers (*p* = 0.049)
Youn et al. [47], 2017 Case-Control	N = 689 Republic of Korea 344 SMC individuals Mean age: 70.0	APOE ε7	Seoul Neuro-Psychological Screening Battery (SNSB)	Only SMC subjects had APOE ε7 variants (heterozygotes ε3/ε7)
Watfa et al. [48], 2010 Cross-Sectional	N = 369, France SMC, hypertensive Mean age: 70.0 ± 6.0	50 vascular- related genes	Cognitive Difficulties Scale of McNair	No association of any of the polymorphisms with cognitive decline

^1^ Subjective memory complaints, ^2^ cognitively normal, ^3^ subjective cognitive decline, ^4^ mild cognitive impairment.

**Table 3:** Polygenic risk scores in individuals with SCD.

Study	Population	Polygenic Risk	SMC ^1^ Estimation	Main Results
Ebenau et al. [49], 2021 Cross-Sectional	N = 829, The Netherlands All SCD ^2^ individuals Mean age: 59.6 ± 8.8	PRS ^3^ including 39 genetic loci related to AD ^4^	Amsterdam Instrumental Activities of Daily Living questionnaire	PRS was associated with amyloid positivity (OR: 1.5)
Sathyan et al. [50], 2019 Case-Control	Ν = 4915, United States 1928 SMC individuals Mean age: 74.8 ± 6.7	PRS including 19 genetic loci related to AD	In-person interview: 2 questions	PRS for AD was not associated with SMC (*p* = 0.407).
Bell et al. [44], 2023 Longitudinal	N = 1555, United States Male adults Mean age: 37.8 ± 2.5		In-person interview: single question	PRS for AD was not associated with SMC

^1^ Subjective memory complaints, ^2^ subjective cognitive decline, ^3^ polygenic risk score, ^4^ Alzheimer’s disease.

## Data Availability

The data that support the findings of this study are available in PubMed/MEDLINE and EMBASE.

## Supplementary Materials

The following supporting information can be downloaded at: https://www.mdpi.com/article/10.3390/cimb46030129/s1, Table S1: Systematic Review inclusion and exclusion Criteria; Table S2: PRISMA 2020 Systematic Review Checklist; Table S3: PRISMA 2020 checklist for abstracts.
